# Heterogeneous trait responses of Páramo plant species and community to experimental warming

**DOI:** 10.1098/rspb.2025.0245

**Published:** 2025-06-18

**Authors:** Carolina Tovar, Sidonie Bellot, Melissa Llerena-Zambrano, Ilia Leitch, Priscila Carpio-Cordero, María Genoveva Granda-Albuja, Jonathan Dario Rondal, Sisimac Duchicela, Antonella Luciana Bernardi, Edison Salazar, Sahr Mian, Eduardo Tejera, Gabriela Echevarría, Francisco Cuesta

**Affiliations:** ^1^ Royal Botanic Gardens, Kew, Richmond, London, UK; ^2^ Grupo de Investigación en Biodiversidad Medio Ambiente y Salud-BIOMAS, Universidad de las Américas, Quito, Pichincha, Ecuador; ^3^ Laboratorios de Investigación, Universidad de las Américas, Quito, Pichincha, Ecuador; ^4^ Department of Geography & Institute of Arctic and Alpine Research, University of Colorado Boulder, Boulder, CO, USA; ^5^ Grupo de Bio-Quimioinformática, Facultad de Ingeniería y Ciencias Aplicadas, Universidad de las Américas, Quito, Pichincha, Ecuador; ^6^ Global Research & Solutions Center, Universidad San Francisco de Quito, Quito, Pichincha, Ecuador

**Keywords:** Páramo, climate change, functional traits, experimental warming, fast growth strategy, slow growth strategy

## Abstract

Understanding the impact of climate change on the functional trait composition (and hence ecosystem functioning) of tropical alpine regions is critical for predicting biodiversity responses. We tested the effects of a decade of warming on the morphological, chemical and genomic traits of Páramo species using open-top chambers (OTCs). We conducted vegetation surveys and collected samples from individuals inside and outside the OTC plots to estimate differences between treatments (warming versus control). Vegetation cover decreased over time in both treatments suggesting a potential decline in soil moisture in our study area. Warming led to a reorganization of the trait space and trait network structure. Species showed a wide range of responses to warming, with significant changes across different trait combinations. Nevertheless, we did not find significant differences in trait values or the direction of change between species whose percentage vegetation cover increased in OTC (or decreased less) over time, compared with control. Community-weighted mean values of plant height, leaf area, leaf dry matter content, genome size, leaf C and P, significantly increased over time only in OTC plots (i.e. traits associated with carbon storage and decomposition). While warming and reduced soil moisture lead to heterogeneous species responses without a clear winning trait strategy, changes at the community level may have important implications for Páramo ecosystem functioning.

## 1. Introduction

Mountain ecosystems have undergone profound changes in climate in the last decades [1], which have led to significant fluctuations in their vegetation. Across mountain ecosystems globally, changes have been recorded not only in plant species richness (e.g [2,3]) and plant range dynamics (e.g. [4,5]), but also in the composition of plant functional traits across communities (e.g. [6,7]). Functional traits of the dominant species are predicted to play a predominant role in ecosystem functioning (according to Grime’s mass ratio hypothesis [8,9]), thus, shifts in the composition of species in a community, which involve the dominant species, are likely to have an impact on ecosystem functioning. However, our understanding of potential shifts in the functional composition of communities in tropical alpine regions due to climate change remains very limited.

A set of rules organize plant form and function globally, which is represented as a trait space (combinations of plant trait values shown in a bi- to multi-dimensional space) [10]. According to these rules, one major axis of variation is defined by a gradient along the size of the plants and their organs, and a second major axis is associated with the leaf economic spectrum, which represents a trade-off between leaf construction investment and growth [10]. Traits associated with low investment in leaf construction are, for example, low leaf dry matter content (LDMC), high specific leaf area (SLA) and high leaf N, characteristics that result in fast growth [11]. Low investment and fast growth are expected to be dominant in plants occupying areas with non-limited resources (i.e. optimal growth temperature, high soil fertility, light and water availability) [12]. In contrast, slow growth is expected when resources are limited (e.g. in high-elevation sites with limited nutrients) as species tend to prioritize survival over growth, which is characterized by an opposite trend in growth-associated traits [13,14].

The trait space defined for alpine species globally also shows that the main axes of variation are related to the leaf economic spectrum (axis 1) and plant size (axis 2) [15]. Nevertheless, differences are observed between the biogeographic realms, with alpine species from Neotropical regions generally showing higher investment in leaf construction than those from Palaearctic or Nearctic regions [15]. Other traits have been less explored, notably leaf chemical composition and genome size, despite the potential of these traits to interact and impact the extent of variation in the major functional traits noted above (e.g. [16–18]). The influence of chemical (e.g. leaf Fe, Na, etc.) and genomic traits (e.g. genome size) in the trait space, has received little attention in alpine species, particularly in tropical regions. More recently, trait network analyses (interdependencies between traits inferred from correlations [19,20]) showed higher connectivity between traits in warmer climates than in colder climates, with implications for resource use [20]. Hub traits, those with a high number of connections to other traits, could also shift due to warming [20]. Thus, trait networks offer a complementary analysis to the trait space to better understand the trait relationships in alpine environments.

Trait values are linked to environmental and resource gradients [12] with some traits being more sensitive to environmental changes (response traits) and others mediating ecosystem functioning (effect traits) [21]. Thus, changes in climate are expected to impact response traits, and, if these overlap with effect traits, also ecosystem functioning. Warming could also positively or negatively impact species fitness such as abundance or survival [5]. While some species may adjust their trait values to better tolerate warming, others may lack this phenotypic plasticity [22]. Both experimental warming and transplant approaches have been used to explore primarily the impact of climate change on the functional composition of alpine communities. For instance, an examination of leaf traits in a transplant experiment from 2000 to 1000 m a.s.l. in the Swiss Alps showed that warming favoured species with a more conservative water use strategy [23]. Experiments using open-top chambers to simulate passive warming have yielded mixed results. For example, in an alpine region of Norway, experimental warming led to a reduction in leaf N concentration and to an increase in the leaf C/N ratio, indicating a shift towards a slower growth strategy [24]. In contrast, similar experiments conducted on the Tibetan plateau demonstrated that warming promoted an increased plant height, greater leaf area and a reduction in [20,24].

The Páramos, located in the northern high Andes, are the most species rich of the tropical alpine regions [25] and have one of the highest net diversification rates of the world’s biodiversity hotspots [26]. These high-mountain ecosystems play a fundamental role in water regulation and thus have an impact on the water cycle (e.g. [27]). Additionally, they contribute to climate change mitigation through the storage of carbon in their biomass and soils (e.g. [28]). Given the observed increase in mean annual temperature in the Andes over the last 30−40 years [29] and the projected increases of up to 4°C in the highest areas (above 4000 m) by 2100 [30], it is of utmost importance to understand how climate change is likely to impact the functional composition of the Páramos’ plant communities and hence their ability to continue to provide key ecosystem services.

Recently, heterogeneous changes in species composition across the high-Andes (with elevation ranging from approx. 650 m a.s.l. in the Argentinian southern Patagonia to >5000 m a.s.l. in the Peruvian southern Andes) have been observed, mostly towards a marked increase of warm-adapted species in locations closer to the equator [2]. This could potentially lead to modifications in functional traits, and hence, a potential shift in how the ecosystem functions. For example, a study using a warming experiment with open-top chambers (OTCs) in the Ecuadorian Páramos evidenced a significant increase in above-ground biomass under warming compared with the control plots [31]. However, the mechanisms behind this in terms of changes in functional traits due to warming were not assessed. This raises the question of whether certain trait values enable species to better cope with warmer conditions. A global study has identified that taller plants, those with lower SLA and higher water use efficiency are plants with advantageous traits in montane grasslands to enhance fitness, either for plant survival, enhanced reproductive output or vegetation biomass [32]. However, whether these findings apply to the Páramos remains to be tested.

Here, using a warming experiment installed in 2012 located at 4200 m a.s.l. in the Yanacocha Reserve, Ecuador, we aim to test the hypothesis that 10 years of passive warming (2012–2022) has led to changes in both the values of functional traits of the dominant species and of the plant community. We have three main objectives: (i) to characterize the functional trait space and trait network, using traits of 40 dominant species and whether trait space and network shift with warming; (ii) to analyse species responses to warming, including changes in traits values and vegetation cover, the latter as a proxy for fitness; and (iii) to compare changes in the functional composition of the plant community over time between OTC and control plots.

## 2. Material and methods

### (a) Study area and experimental design

The study area is located in the Páramos of the Yanacocha Reserve, Ecuador, at 4200 m a.s.l. (0.13518° S, 78.57458° W) (electronic supplementary material, figure S1). The passive warming experiment was installed in 2012 and consists of five parallel monitoring blocks, each of them containing 40 plots (each of area 1 m^2^) delimited for different treatments. Plots assigned to a warming treatment were surrounded by a 3 × 3 m hexagonal OTC. Control plots and those under warming treatment (OTC plots) were randomly chosen across the five blocks. Our analyses are based on the data from 17 OTC plots and 20 control plots.

**Figure 1.**
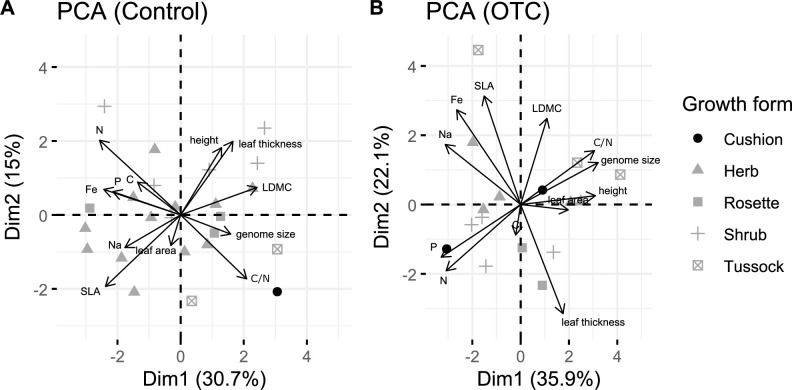
Species trait space is shown in PCA biplots from samples collected in (A) control and (B) OTC conditions. SLA, specific leaf area; LDMC, leaf dry matter content.

In the region, mean annual precipitation is around 1600 mm [33] although it varies across the year with one main dry period, from July to September and mean monthly temperature is 6.5−8°C [34]. Measurements from loggers installed for the experiment indicate differences between control and OTC plots were more significant for maximum daily temperatures during the dry season (OTC = 28°C, control = 25.7°C) and maximum night temperatures across the year (OTC = 9.7°C, control = 8.4°C) [31].

### (b) Field data collection

#### (i) Vegetation surveys

Before installing the OTCs, a baseline survey was conducted in 2012, recording vegetation cover (%) of each seed plant species per plot. A further five vegetation surveys were conducted on the control and OTC plots in 2013, 2014, 2016 (a few plots for this survey were measured in December 2015), 2017 and 2019. The OTCs of five warming plots were blown away by the wind after the 2017 survey, therefore, for 2019, we used the data from the remaining OTCs (electronic supplementary material, table S1). A total of 71 taxa were recorded in the selected plots but we chose to work with the 40 most dominant species based on percentage vegetation cover (see electronic supplementary material, table S2 and appendix S1 for details on species selection).

#### (ii) Collection of leaves for trait analysis

All trait data were collected in June 2022. To measure the morphological traits for each species whenever possible (i.e. we were limited by the number of individuals present in OTCs), we collected five leaves each from three individuals inside the OTC plots and from three individuals outside them. We also measured the vegetative plant height of 1 to 10 individuals per treatment (OTC mean = 6, control mean = 8). For chemical traits, due to the small size of most species, collecting enough material for analysis (see electronic supplementary material, appendix S1) was challenging and thus, for a given treatment (warming or control) and for each species, we pooled the leaves from more than one individual together. Finally, for the genome size analyses, we collected leaf material from one individual per species and stored them in a plastic bag with a wet tissue to preserve them until analysed. Given the variable availability of plant material, it was not possible to collect samples for all traits and all 40 species (electronic supplementary material, table S2 shows which traits were analysed for each species).

### (c) Laboratory analyses

Samples for morphological and chemical analyses were taken to the laboratories of the Universidad de las Américas in Ecuador where the following morphological traits were measured: (i) leaf area (cm^2^), (ii) leaf blade thickness (mm), (iii) specific leaf area (SLA in cm^2^ g^−1^), which is the fresh leaf area divided by its dry mass, and (iv) leaf dry matter content (LDMC in mg g^−1^), which is the dry leaf mass divided by the fresh leaf mass (see processing details in electronic supplementary material, appendix S1).

Samples for characterizing the chemical traits were processed (electronic supplementary material, appendix S1) to estimate: (i) the amounts of macro- (i.e., K, P, Mg, Ca in ppm) and (ii) micro-nutrients (i.e. Al, B, Fe, Na in ppm) in the leaves, and (iii) the leaf C and N content (%) and C/N ratio. Finally, the genome size (number of base pairs in the DNA in gigabases per unreplicated gametic nucleus (i.e. the 1C-value), Gb/1C) of each species was estimated at the Royal Botanic Gardens, Kew, by propidium iodide flow cytometry (see details in electronic supplementary material, appendix S1).

### (d) Statistical analyses

#### (i) Trait values treatment

The analyses of intraspecific trait variability and species responses were based solely on the morphological traits because of the availability of a sufficient number of replicates per species, while for chemical and genomic traits this was not possible. Analyses related to trait space, trait networks and community weighted means (CWM) used morphological, chemical and genomic traits. Prior to these later analyses, we averaged the values per trait per treatment per species for morphological traits.

#### (ii) Changes in trait space, intraspecific trait variability and trait networks due to warming

We conducted a principal component analysis (PCA) to characterize the trait space of the studied species for each treatment and to identify the main axes of variation. All traits were log-transformed, centred and scaled before analysis. We first performed a correlation analysis for all chemical variables due to the high number of measured variables and selected the following uncorrelated ones: P, Fe, C, N, Na, C/N. As the PCA requires data from across all trait values, we had different sample numbers for each PCA (electronic supplementary material, table S2). We also performed one more PCA using samples of both treatments together to visualize potential changes in the area of the trait space and tested for significant differences in trait composition using a PERMANOVA.

We estimated the intraspecific variability for each species only for the morphological traits using the coefficient of variation (CV) values for both control and OTC. We used a Games–Howell post hoc test to look for significant differences between the traits analysed across species.

We also built separate trait networks using the Igraph R package [35] for both control and OTC samples based on Pearson correlations between all morphological traits, genome size and the selected chemical traits from the PCA (see above), after log-transforming them. Traits were treated as nodes and the correlation values as edges in each network. From these analyses, we estimated two metrics: (i) modularity (whether chemical, morphological and genomic traits were more connected (low modularity) or less connected (higher modularity) and (ii) the degree of centrality for each trait (where the trait with the highest number of edges connected to other traits is considered a hub trait) using the function ‘degree’ [19,36].

#### (iii) Species responses to passive warming

We first estimated changes in vegetation cover per species between 2012 and 2019 per treatment for 39 of our 40 species (*Astragalus geminiflorus* was not recorded in OTC plots) as a proxy of change in fitness. Subsequently, we analysed changes in trait values and trends for each species.

We used linear mixed effects models with the percentage vegetation cover of each species per plot as a dependent variable, the survey year treated as a fixed independent variable and the plot as a random effect. Given that vegetation cover values ranged between 0 and 100 (%), we applied a beta distribution model, with a square-root transformation of the vegetation cover data. Based on the model outputs, we identified three different types of responses: (i) species that lost more vegetation cover over time under warming than in control conditions (negative fitness response to warming), (ii) species that increased in vegetation cover over time under warming in comparison with control or that lost less than in the control plots (positive fitness response), and (iii) species with no significant difference in vegetation cover trends over time between treatments (neutral response). Additionally, we used a Wilcoxon signed-rank test for paired data to compare the slopes of the species’ regressions between control and OTC plots.

We then ran three tests to explore differences in morphological traits between treatments (i.e. control versus warming). We first tested for intraspecific differences in trait values between samples collected inside and outside the OTC plots using a Wilcoxon rank-sum test. We then tested differences in trait trends by grouping species based on how their traits responded to warming (i.e. significantly higher in OTC, significantly lower and no significant change) using a divisive cluster based on a Gower dissimilarity matrix in the cluster R package [37] and determined the optimal number of clusters using the silhouette method. Finally, we used a phylo-ANOVA [38] to test for differences in trait values between species with positive, neutral and negative fitness responses to warming (as determined by the vegetation cover analysis), while accounting for phylogenetic relationships (see below), which could obscure the results due to the tendency for closely related species to resemble each other’s traits more than by chance.

To infer phylogenetic relationships between the 40 studied species we used DNA sequence data publicly available in GenBank. The four most frequently sequenced genetic regions among the study species were the nuclear ribosomal internal transcribed spacers (ITS1 and ITS2) regions flanking the 5.8S ribosomal RNA region), the plastid coding genes *matK* and *rbcL*, and the plastid intergenic spacer *trnL-trnF*. These regions were downloaded for all species for which they were available and if the species was missing, we replaced it (see details in electronic supplementary materials, appendix S1 and table S3). A separate sequence alignment was performed for each genetic region (see details in electronic supplementary material, appendix S1). After preliminary tests showing the lack of strongly supported conflicts between phylogenies obtained from each region separately, the four alignments were concatenated in a single matrix. IQ-TREE v 1.6.12 was used to select the best nucleotide substitution model [39] for each individual region and to infer the species phylogeny based on the concatenated matrix, unlinking branch lengths between partitions. A thousand ultrafast bootstrap replicates [40] were performed to assess the support of each clade in the resulting maximum likelihood tree.

#### (iv) Community responses to warming

We first analysed trends in total vegetation cover per plot over time for surveys conducted between 2012 and 2019. Then we *estimate*d non-parametric bootstrapped CWMs and the CWM variance for each trait per plot per survey for morphological, chemical and genomic traits using the ‘traitstrap’ package [41]. This approach estimates the mean based on bootstrapped trait distributions. In all cases, we used the percentage vegetation cover of each species to weight the trait value in each control and OTC plot per survey. We only used the plots for which sufficient trait data existed (see details in electronic supplementary material, appendix S1). We estimated the contribution of each species to the CWM of each plot per survey as the proportion of the species cover in relation to the total vegetation cover of all seed plant species present in each plot. Finally, we used a Wilcoxon rank-sum test to assess differences at the community level (CWM trait values) between the baseline survey (2012) and the last survey (2019) in both the control and OTC plots.

## 3. Results

### (a) Trait space, intraspecific variability and trait networks for Páramo species

The distribution of morphological trait values under control conditions was skewed towards low values (electronic supplementary material, figure S2), and median values across species were 6 cm (plant height), 0.65 cm^2^ (leaf area), 0.29 mm (leaf thickness), 200 mg g^−1^ (LDMC) and 210 cm^2 ^g^−1^ (SLA). Most trait distributions of chemical trait values, except leaf C, were skewed towards low values, but values were spread across a wide range using control samples (electronic supplementary materials, figure S2 and table S4). For OTC conditions, trait distributions were slightly skewed towards the right in comparison to control conditions for plant height, leaf C, C/N, P and Na. From a genomic trait perspective, genome sizes ranged 24-fold from 0.5 Gb/1C in *Bartsia stricta* to 12.0 Gb/1C for *Monticalia peruviana* (electronic supplementary material, table S4) with a median of 2.3 Gb/1C.

**Figure 2.**
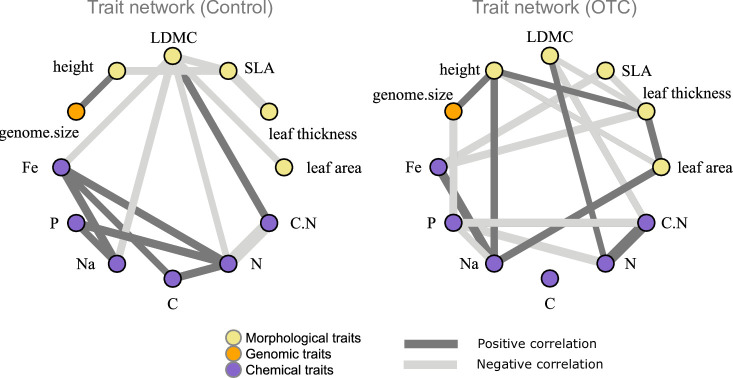
Trait networks for samples collected outside the OTCs (Control) and inside the OTC plots. Lines denote significant trait-trait correlations (*p*‐value < 0.05). ( LDMC, leaf dry matter content; SLA, specific leaf area).

Among all morphological traits, plant height showed the highest intraspecific variation (median CV = 51% across species) while leaf thickness had the lowest (15%) (electronic supplementary material, figure S3A) for both control and OTC samples.

**Figure 3.**
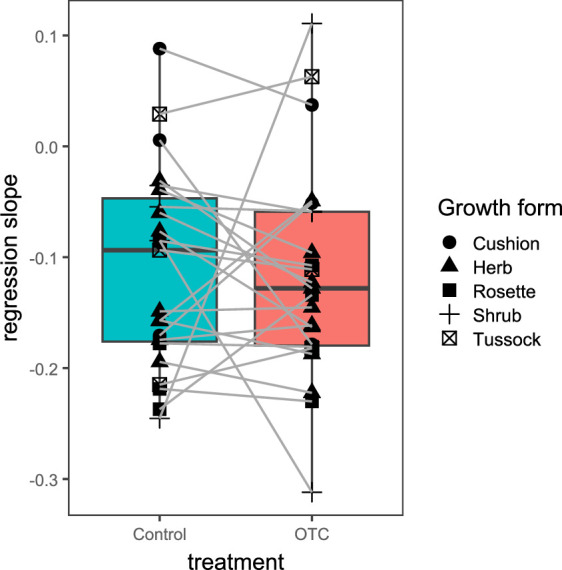
Boxplots comparing species trends in vegetation cover change over time between control and OTC. Each line represents one species where the initial value on the left (control) represents the slope of the regression fitted for vegetation changes over time for control samples only, and the end of the line represents the slope of the regression for OTC samples only. Positive slopes in the *y*-axis indicate an increase in vegetation cover over time while negative values indicate the opposite.

When all chemical, genomic and morphological traits were grouped to analyse the species trait space under control conditions, the variation explained by the first two axes of the PCA biplot was lower for control (45.7%) than for OTC samples (58%) (figure 1A,B). Under control conditions, the first axis showed a gradient from low leaf construction investment (i.e. high leaf nutrient concentrations of N, Fe and P) to high investment (high C/N and high LDMC; figure 1A; electronic supplementary material, table S5). The second axis was also related to construction investment, from high SLA and thinner leaves to thicker leaves, higher N, and, with less contribution, taller plants. In contrast, the OTC PCA shows that plant size defines the first axis of variation, from small plants with high leaf nutrient concentrations (P, N) to taller plants with bigger genome sizes and higher leaf C/N (figure 1B). The second axis is related to the leaf construction investment, showing a gradient from thicker leaves to thinner leaves, high SLA and LDMC and high leaf nutrient concentrations. On comparing the trait space of both treatments in one PCA, we observed the trait space area of OTC samples is a bit larger than that of the control samples (electronic supplementary material, figure S4); however, no significant differences are found in trait composition using a PERMANOVA (electronic supplementary material, table S6).

**Figure 4.**
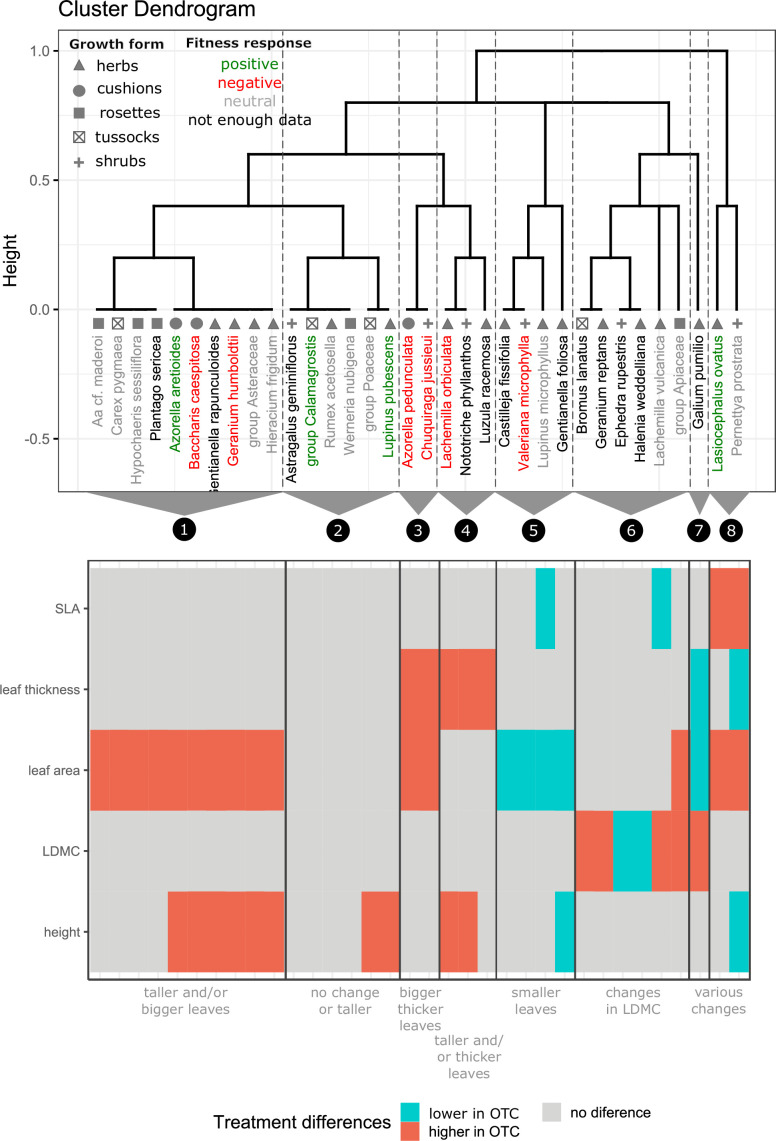
Dendrogram showing the clustering of species into eight groups based on their trait responses to warming (OTC) in comparison to control. Top figure shows the dendrogram with the eight groups separated by dotted lines, and species with a positive, negative or neutral response to warming based on changes in percentage vegetation cover under warming labelled in green, red or grey, respectively). The bottom part of the figure shows the changes (or not) of individual traits for each species as a heatmap but organized according to the dendrogram. The main combinations of traits which characterize each group are described at the bottom of the figure.

Trait coordination in the trait network changed with warming (figure 2). In the trait network for the control samples, none of the morphological traits showed significant correlations with chemical traits (modularity = 0.20), except for LDMC. In contrast, in the OTC trait network, there were more significant correlations between these two groups of traits (modularity = 0.11). In addition, there was a shift in the hub trait between treatments, with LDMC being the hub trait (degree = 0.50) in the control network, while leaf thickness was the hub trait in the OTC network (degree = 0.41). Finally, the direction of some of the correlations (for instance between leaf P, leaf Na and leaf N) also changed between the control and OTC networks.

#### (i) Species response to passive warming

Trends in vegetation cover over time showed significant differences between control and OTC for 11 species of which five showed a positive fitness response to warming and six a negative response (electronic supplementary material, table S7, examples of a few species are shown in electronic supplementary material, figure S5). Twelve species had a neutral response, while for 17 species the models did not converge due to small sample sizes (electronic supplementary material, table S7). When comparing all species’ regression slopes between control and OTC, we did not find significant differences (*p* = 0.41, figure 3).

**Figure 5.**
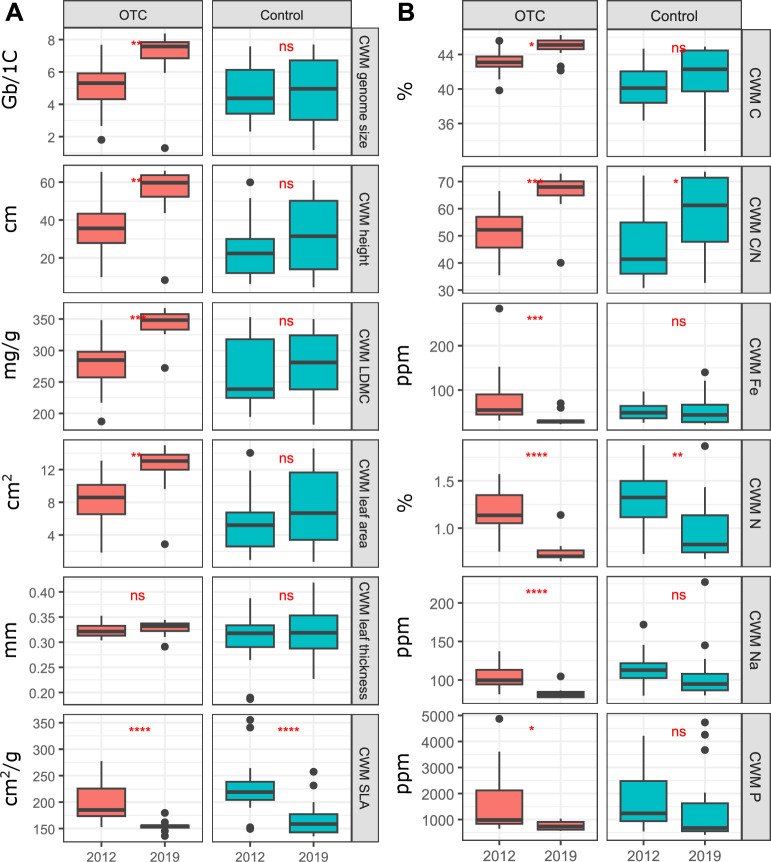
Comparison between the 2012 baseline survey and the 2019 survey in the CWM values for (A) the morphological and genomic traits analysed in the control and OTC plots and (B) the chemical traits analysed in the control and OTC plots. LDMC, leaf dry matter content; SLA, specific leaf area. Differences between control and OTC were tested with a Wilcox test (*p*‐values: *<0.05; **<0.01; ***<0.001 ).

Our cluster analysis of species based on their morphological trait responses, showed eight groups (figure 4). The most common trait response to warming was an increase in height and/or leaf area (*n* = 10 species), followed by changes in LDMC (either higher or lower; *n* = 6). Other response strategies included developing thicker leaves or growing taller plus having thicker leaves. Generally, there was no consistent association between trait responses and fitness responses to warming or growth form.

When visually assessing potential phylogenetic structure in trait responses, we found that all Apiaceae species (*n* = 3) and all Asteraceae species (except for *Werneria nubigena*, *n* = 7) had significantly larger leaf areas in warming than in control conditions (electronic supplementary material, figure S6). Nevertheless, species’ fitness responses to warming did not show any obvious phylogenetic structure (electronic supplementary material, figure S6).

Finally, we found no significant differences in morphological trait values between species with positive and negative fitness responses either using control or OTC samples (electronic supplementary material, figure S7 and table S8).

### (b) Community response to warming

Overall, there was a decrease in the total vegetation cover (%) of the plots over time, regardless of the treatment (electronic supplementary material, figure 8A). However, when pooling data across all plots and surveys we found that OTC plots had a significantly higher vegetation cover than control plots (*p* = 0.01, electronic supplementary material, figure 8B).

We found a significant increase in the CWM values for LDMC, leaf area, plant height and genome size in OTC plots between 2012 and 2019, while no significant change was detected in control plots (figure 5). Similarly, we observed significant increases in CWM values for leaf C and P concentrations in the OTC plots over time while no significant change or a significant decrease, respectively, were observed in the control plots. At the same time, we also observed that most of the CWM variance of morphological, genomic, and chemical traits significantly decreases over time in OTC plots while hardly any significant difference was found in OTC plots (electronic supplementary material, figure S9).

Species contributions to OTC CWM estimates showed that *Chuquiraga jussieui* (with a negative fitness response) and the *Calamagrostis* group (with a positive fitness response) were the primary contributors to CWM values (electronic supplementary material, figure S10). In contrast, the influence of other species in the CWM estimates, such as *Azorella pedunculata*, declined over time in OTC plots, reducing their contribution to CWM estimates.

## 4. Discussion

Our main results show that a decade of passive warming had shifted the trait space of Páramo plant species and led to heterogeneous species responses with different trait combinations and different fitness changes. At the community level, changes were observed in traits associated with more conservative strategies. However, it is important to note that vegetation cover declined over time in both the OTC and control plots, suggesting that reduced soil moisture probably played a role during our study period. Long-term analysis (1895−2015) of instrumental data from the Quito region, located approximately 10 km from our study area at 2800 m a.s.l., indicate an increase in the frequency and intensity of droughts since 1950 [42]. While we cannot confirm the occurrence of a drought event during the study period, a reduction in precipitation or a prolonged dry season could have contributed to a decrease in soil moisture, ultimately driving the observed decline in vegetation cover. These contextual factors should be considered when interpreting our results.

### (a) Species trait space shifts under warming

Two main axes organize the form and function of plants globally, the leaf economic spectrum (from high-to-low leaf construction costs) and the size axis (from smaller to bigger plants or plants with bigger organs) [10]. While a similar pattern is found for alpine species globally [15], neotropical alpine species, such as those in the Páramo, have limited variation along the size axis compared with species from other alpine regions [15]. Our PCA results indicate that species in our study area, under control conditions, follow ecological strategies primarily structured by the leaf economic spectrum on both axes of variation (figure 1). This finding aligns with previous research conducted in the Colombian Páramos [43]. The role of chemical traits (i.e. leaf nutrient concentrations) is prominent in the first axis distinguishing between species with fast growth, which require high foliar concentrations of leaf nutrients, such as N, P and Fe [17,44], and species with slower rates of growth characterized by traits such as high LDMC [11]. Leaf thickness dominated the second axis, which has been linked with structural resistance to physical damage [45]. Warming, however, increased the importance of (i) size-related traits in the first axis where taller plants were linked to bigger genomes, and (ii) leaf thickness in the second axis. Overall, warming was shown to reorganize the trait space departing from what was expected for the trait space of neotropical alpine species [15] despite the size of the trait space does not differ between warming and control (electronic supplementary material, figure S4).

We also observed high intraspecific variation for some traits despite our study area being limited to one location. Plant height had the largest CV. This most likely reflects competition for light as species in equatorial alpine environments require high carbon gain efficiency to withstand the extreme night cold conditions [46]. Leaf thickness had the lowest variation which was significantly different from all the other traits analysed (electronic supplementary material, figure S3). Despite leaf thickness ranging from 0.13 mm (*Galium pumilo*) to 0.82 mm (*Werneria nubigena*), there was low intraspecific variation. As this trait has been linked to solar radiation, wind resistance and leaf turgor maintenance [44,47] perhaps the cost of changing leaf thickness is high in the cold tropical alpine environments of the Páramos.

### (b) Trait coordination is higher under warming

Trait coordination analysis revealed greater independence (i.e. higher modularity) between trait groups (i.e. morphological, chemical and genomic) in the network built from samples collected outside the OTCs than that built from samples collected inside the OTCs (figure 2). This suggests that species in colder environments, such as our control conditions, may benefit from maintaining greater independence between trait groups, potentially enhancing their flexibility to adapt and manage risks associated with cold stress [19,48]. After a decade of exposure to warmer conditions, the observed increase in coordination between leaf nutrient levels and leaf morphology likely reflects a shift towards more efficient resource use in warmer environments, where both productivity and competition are expected to increase [49]. This aligns with findings from a previous study in the area, which reported higher above ground biomass in the warming plots compared with control plots [31] despite the hypothesized decrease in soil moisture during the study period. Thus, conditions in the OTC seem to favour higher connectivity between traits, potentially influencing ecosystem functions such as above ground biomass allocation. Additionally, we observed a shift from LDMC being the hub trait in control conditions to leaf thickness under warming conditions, whose importance also increased in the warming trait space (figure 1). This suggests that warming not only altered the overall trait space but also reshaped the relationships among traits.

### (c) Heterogeneous species responses to warming

Individual species’ responses to warming were heterogeneous both in terms of their fitness responses (i.e. change in vegetation cover) and their trait responses (e.g. leaf N, SLA, leaf thickness, etc.).

Half of the species for which the fitness response models converged showed either a positive (26%) or negative (30%) fitness response to warming while the other half had a neutral response. (figure 3, electronic supplementary material, table S7). This heterogeneous response mirrors results synthesised from warming experiments and long-term monitoring in the Artic but where ‘no change’ in abundance (neutral) was the most common response [50]. The few species whose abundance increased over time in the Arctic were shrubs and graminoids [50]. In our study area group *Calamagrostis* (graminoid) and *Monticalia peruviana* (shrub) were among the 5 species with a positive fitness response. Longer time series could increase the number of species with a fitness response model and give a clearer pattern of vegetation cover responses to warming.

Trait responses were also heterogeneous where at least half of the species showed significant differences in more than one trait when comparing control and OTC samples (figure 4). The most common trait response to warming was an increase in leaf area, often accompanied by taller individuals, reflecting a greater investment in size [10], which is consistent with other warming experiments in the Artic [51]. Increasing leaf area enables higher heat loss and reduces the risk of severe heat stress when temperatures rise, this is an advantage for alpine plants as typically they have higher leaf temperatures than the air to withstand the cold alpine temperatures [52].

However, trait responses were also seen to be independent of fitness responses. Thus, while we expected similar trait responses or trait values among species with a positive fitness response to warming [32], this was not the case (figure 4; electronic supplementary material, figure S7). For example, while two of the species with positive fitness responses (*Azorella aretioides* and *Lupinus pubescens*) had taller individuals under warming, two other taxa that showed neutral fitness responses to warming (*Lachemilla vulcanica* and group Apiaceae), also had taller individuals under warming. Similarly, all three cushion species showed increases in their leaf area under warming but, while *Azorella aretioides* showed a positive fitness response the other two (i.e. *Baccharis caespitosa* and *Azorella pedunculata*) showed a negative response.

One explanation for the lack of a clear set of winning traits is that we could have missed key traits in our analysis such as below ground root or clonal traits [20,53]. Another possibility is that, although our species seem plastic enough to respond in different ways to the moderated experimental warming, perhaps a longer experimental period or higher warming rates are needed to see if trait responses converge over time. Another alternative is that phylogenetic conservatism plays a role by differently constraining the trait response of different clades. For example, most species in Asteraceae and Apiaceae had significantly higher leaf areas in warming conditions. While our study highlights the plasticity of these species, which is also reflected in their high thermal niche breadths [54]*,* it also shows that not all traits will necessarily respond to environmental filtering.

### (d) Community increases conservative strategies

Community responses to warming—and a potential decrease in soil moisture—has led to an increase in conservative strategies over time in our study area. For example, vegetation cover of species with higher investment in leaf construction (e.g. high LDMC) significantly increased in OTC plots between 2012 and 2019 but not in control plots. Group *Calamagrostis*, the most dominant taxa in our study area who has medium-high values of LDMC, increased its importance in the community composition over time in the OTC plots (electronic supplementary material, figure S10), thus probably contributing to the increase in CWM values in the warming treatment. The overall increase in the CWM value for LDMC was expected to lead to a decrease in leaf nutrients because LDMC has been shown to negatively correlate with the ability of plants to retain nutrients (figure 1) [44]. Indeed, except for CWM leaf P and C, CWM of leaf Fe, Na and N decreased under warming. High LDMC values have also been associated with a stress tolerant strategy [55]. While the main stress in the Páramo comes from the low temperatures, high LDMC values have also been associated with resistance to drought and herbivory [56].

The increase in the CWM genome size under warming, which has also been associated with a conservative strategy [57], is likely underpinned by the large contribution to the community vegetation cover of the group *Calamagrostis* (electronic supplementary material, figure S10) which has one of the largest genomes among our studied species (i.e. 8.5 Gb/1C). Such a finding is perhaps surprising as numerous studies have suggested that species with bigger genomes may be less competitive in a plant community due to their more constrained trait space [16]. Such constraints arise from the biophysical impact of genome size which sets the minimum values for cell size and cell cycle duration (e.g [16]), nutrient demands (e.g [57]) and cell packing densities [58] that in turn, can impact photosynthetic capacity, and hence the ability to generate biomass [18,58]. While other trait adaptations may also have contributed to the ability of group *Calamagrostis* to compete successfully in the warming treatment, it is possible that the presence of polyploidy in this group may have enhanced its competitive success despite the biophysical constraints imposed by its large genome. Species with larger genomes that have arisen from polyploidy may be more competitive than diploid species with similar genome sizes due to their potentially greater genetic diversity and hence adaptability in the face of environmental change [59,60]. In addition, polyploids are often observed to have larger leaves, seeds, stomata, in part due to their larger cells and potential to undergo more rapid growth through cell expansion [61]. While the ploidy level of the species comprising group *Calamagrostis* is currently unknown, polyploidy is known to be common in the genus and hence this might contribute to explaining why *Calamagrostis* is able to dominate the vegetation and hence results in the increased CWM genome size observed in our study. The result is likely to have also been compounded by the decrease in percentage vegetation cover under warming over time of other originally more dominant species with smaller genomes, such as *Azorella pedunculata* (i.e. 0.91 Gb/1C).

In addition to the increase in the CWM of LDMC and genome size, we also observed an increased representation of taller plants and bigger leaves in the community. Across several warming experiments in the Artic using OTCs, only CWM plant height increased [22], suggesting that the combination of warming and potentially reduced soil moisture may trigger more functional changes than warming alone. Despite the heterogeneous trait responses across species, the functional composition of our Páramo community is consistently changing towards more conservative strategies and bigger plants.

### (e) Implications for ecosystem functioning

Changes in CWM in the OTC plots, driven by the dominant species, will probably influence the ecosystem functioning of the Páramos according to the mass ratio hypothesis [8,9]. The observed increase in CWM for LDMC under warming could potentially slow decomposition and mineralisation [62] in our Páramo community. Although a study in the Colombian Páramos did not show changes in decomposition after three years of warming using OTCs [63], it is likely that a longer period (such as the 10-year period in our study area) could affect it. A higher LDMC together with bigger leaves arising from warming could create more flammable plant material [62]. Páramo areas other than our study site, where human fires are common [64], could thus potentially see an increase in fire risk under the ongoing warming. We also observed taller plants in the warming treatment, which most likely was the main driver of the observed higher aboveground biomass in OTC plots in comparison to control plots in a previous study [31]. Other ecosystem functioning such as protein content (reflected in leaf N) seemed less affected by the warming as it decreased in both OTC and control plots. Overall, while our findings on functional traits suggest potential changes in ecosystem processes, the concurrent decline in total vegetation cover over time should prompt caution in predicting long-term impacts.

Our ten-year experiment suggests that warming, and potentially drier conditions in the Páramos, will probably lead to heterogeneous responses for species. While species are able to respond through changes in their functional traits, but without a clear winning strategy, changes in community responses are indeed likely to impact key ecosystem functioning such as biomass accumulation and leaf litter decomposition.

## Data Availability

Data and scripts are available at Dryad [65]. Supplementary material is available online [66].
